# Short-term outcomes of total knee arthroplasty performed with and without a tourniquet

**DOI:** 10.1051/sicotj/2021019

**Published:** 2021-03-22

**Authors:** Mihai Grigoras, Oliver Boughton, May Cleary, Paul McKenna, Fiachra E. Rowan

**Affiliations:** 1 Department of Trauma and Orthopaedic Surgery, University Hospital Waterford X91 ER8E Waterford Ireland; 2 Kilcreene Regional Orthopaedic Hospital R95 DK07 Kilkenny Ireland

**Keywords:** Knee surgery, Orthopaedics, Arthroplasty, Tourniquet, Total knee arthroplasty

## Abstract

*Introduction*: Not using a tourniquet could improve early postoperative pain, range of motion (ROM), length of stay (LOS), and thromboembolic risk in patients undergoing total knee arthroplasty (TKA). Our aim was to compare these factors, intraoperative blood loss, and gender-related outcomes in patients undergoing primary TKA with or without a tourniquet. *Methods*: We performed a retrospective cohort study of 97 patients undergoing TKA with or without tourniquet from 2018 to 2020. Revisions and bilateral TKAs were excluded. Blood loss was estimated using a validated formula. Postoperative pain was tested using the visual analogue scale (VAS). ROM and quadriceps lag were assessed by a physiotherapist on a postoperative day 2 and discharge. The index of suspicion for a thromboembolic event was defined as the number of embolic-related investigations ordered in the first 6 months post-surgery. The Shapiro–Wilk test was used to assess the distribution of the data, Mann–Whitney for the continuous variables, and Fischer’s test for the categorical ones. *Results and Discussion*: There was a significant difference in blood loss. The non-tourniquet group lost on average 32% more blood (1291 mL vs. 878 mL, *p*<0.001 two-tailed). We found no difference in pain, ROM, LOS, and quadriceps lag on day 2 and at discharge. There was one thromboembolic event in the tourniquet group, but the thromboembolic index of suspicion did not differ (*p*=0.53). With tourniquet use, women had a significantly lower day 2 maximum flexion than men (71.56° vs. 84.67°, *p*=0.02). In this retrospective cohort study, the results suggest that tourniquet use is associated with lower blood loss and similar postoperative pain, range of motion, quadriceps lag, length of stay, and thromboembolic risk. There might be some differences between how men and women tolerate a tourniquet, with women having worse short-term outcomes compared to men.

## Introduction

Total knee arthroplasty (TKA) is an effective means for treating end-stage osteoarthritis of the knee. According to an American Association of Hip and Knee Surgeons poll and the UK National Joint Registry, most surgeons prefer to use a tourniquet at some point during TKA, but the benefit to risk ratio is still debated in the literature [1, 2]. There is conflicting evidence regarding pain, functional outcome, blood loss from heterogeneous samples of tourniquet use [3–8]. Advocates of tourniquet use cite better visualisation of the surgical field, better cement interdigitation, and overall less blood loss [9–11]. Conversely, tourniquet use might relate to increased pain, limb ischaemia, delayed range of motion (ROM) and quadriceps function recovery, accelerated quadriceps sarcopenia in the elderly, more thromboembolic events, increased length of stay (LOS), and a higher hospital readmission rate [7, 12–14]. More so, the literature is even more scarce and divisive with regards to how tourniquet use might functionally affect genders differently [15, 16]. The primary aim of our study was to determine the effects of tourniquet use during primary TKA on early postoperative functional outcomes, blood loss, and thromboembolic events. The secondary aim was to assess whether tourniquet use affects genders differently. As there is still no consensus on how tourniquet use might affect the outcomes, our study offers a more standardised approach where the tourniquet is either used from the start or not.

## Materials and methods

**Figure 1:**
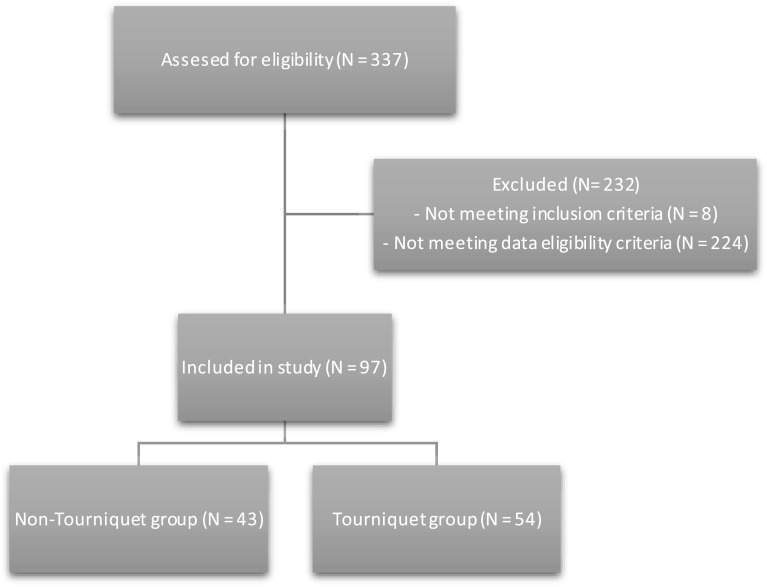
Study algorithm.

This retrospective cohort study was conducted at an academic university hospital that is part of a national total joint registry with prospective follow-up. Institutional approval for the study was received. We included 97 patients with a mean age of 68years having end-stage knee osteoarthritis. The patient demographics are presented in Table 1. To settle on this cohort, we reviewed all TKAs performed at our institution between 2018 and 2020 by two orthopaedic surgeons and 337 TKAs were identified. We excluded 8 cases that underwent bilateral TKAs, revisions, or complex primaries. We defined data eligibility criteria as follows: day 1 postoperative pain scores documented as a Visual analog scale (VAS) – 0 being no pain and 10 being the worst, day 2 postoperative and on discharge knee ROM and quadriceps lag measured by a physiotherapist with a goniometer, blood work was taken perioperatively and history of postoperative blood transfusions. Only 97 patients matched these criteria and had a data completion rate of over 95%. We created two cohorts on account of whether or not the tourniquet was used from the start (43 in the non-tourniquet group and 54 in the tourniquet one) – see Figure 1. One surgeon always used a tourniquet from the start while the other always avoided it. When a tourniquet was used, it was inflated to 100mmHg over systolic blood pressure.

**Table 1:** Patient demographics.

	Tourniquet	No tourniquet	*P* value
Total number of patients	54	43	
Sex			0.08
Male	22	25	
Female	32	18	
Age (years)	70.60 (71.13; 66.89–75.51)	66.42 (67.10; 59.43–72.73)	0.02*
BMI (kg/m^2^)	32.24 (31.85; 29.08–34.40)	32.54 (31.40; 26.51–36.25)	0.82

The data are presented as the mean (median; IQR), where IQR is the interquartile range.

* Marks a significant difference between groups.

All operations were performed under spinal anesthesia. A standard, midline incision, and medial parapatellar approach were employed for all the operations. The knee replacements inserted without a tourniquet were Legion TKRs (Smith & Nephew, Watford, United Kingdom), and the knee replacement implants inserted when using a tourniquet were Triathlon TKRs (Stryker, Michigan, USA). Both implants were cruciate-sacrificing. Traditional, intramedullary femoral instrumentation and extramedullary tibial guides were used. Both surgeons administered 3g of tranexamic acid topically once the fascia was closed, while anaesthesia simultaneously gave 3g IV. The postoperative care included multimodal analgesia as a combination of acetaminophen, nonsteroidal anti-inflammatories, and opioid derivatives. Patients were encouraged to move the surgical limb immediately and formal physiotherapy-led rehabilitation was commenced on the first post-operative day up until and including the discharge day. Blood loss was estimated using the following method [17]:

*Step 1*: Calculate the patient’s blood volume (PBV),

$$\mathrm{PBV}=\left(k1\times h^{3}\right)+\left(k2\times w\right)+k3,$$

where PBV=patient’s blood volume in litres (L), *h*=height in metres (m), *w*=weight in kilograms (kg), *k*1=0.3669 for men and 0.3561 for women, *k*2=0.03219 for men and 0.03308 for women, *k*3=0.6041 for men and 0.1833 for women. PVB is then converted to millilitres.

*Step 2*: Calculate the volume of red blood cell loss (RBC),

$$\mathrm{RBC}=\mathrm{PBV}\times \left({\mathrm{Hct}}_{\mathrm{preop}}-{\mathrm{Hct}}_{\mathrm{postop}}\right),$$

where RBC=red blood cell volume loss in millilitres (mL), Hct_preop_=preoperative haematocrit, Hct_postop_=postoperative haematocrit.

*Step 3*: Convert red blood cell volume loss to total blood volume loss,

$$\mathrm{Total}\mathrm{blood}\mathrm{volume}\mathrm{loss}=\frac{\mathrm{RBC}}{{\mathrm{Hct}}_{\mathrm{avg}}}+\frac{\mathrm{Number}\mathrm{of}\mathrm{units}\mathrm{of}\mathrm{blood}\mathrm{transfused}}{{\mathrm{Hct}}_{\mathrm{avg}}},$$

where Hct_avg_=(Hct_preop_+Hct_postop_)/2. The rationale is that to calculate the total volume of blood loss, the RBC must be divided by the average hematocrit and the transfused blood must be taken into account. An average unit of blood contains 280mL with a Hct of 0.6 which is the red cell equivalent of 168mL with a Hct of 1. Because this is diluted by the patient’s blood volume, the value must in turn be divided by the average hematocrit.

A search of the National Integrated Medical Imaging System (NIMIS; McKesson, Texas, USA) was performed for any investigation related to a thromboembolic event (lower limb venous ultrasound, pulmonary CT angiography, and CT brain) ordered in the first 6 months post-surgery and the outcome. The NIMIS system is a nationwide picture archiving system installed in every academic hospital and can be accessed from any hospital. The index of suspicion for a thromboembolic event in each group was defined as the total number of such investigations ordered, implying that the clinical picture was enough to warrant the clinician to order these costly procedures.

The Shapiro–Wilk test was used to assess the distribution of our data. As the data were not normally distributed, the Mann–Whitney test was conducted for the continuous variables and the Fisher exact test was employed for the categorical ones, with significance set at *p*≤0.05. All analyses were performed using the Statistical Package for the Social Sciences (SPSS) version 24 (IBM).

## Results

### Primary aim

The tourniquet group had significantly less calculated perioperative blood loss (878.2mL) compared to the no tourniquet group (1291.9mL), with *p*<0.001 (Table 2). There was no difference in the self-reported postoperative day 1 pain scores between groups (4.50 in the tourniquet group vs. 4.63 in the non-tourniquet group, *p*=0.85). Neither day 2, nor at discharge knee, ROM and quadriceps lag differed between cohorts. The LOS was also similar between groups (4.57 days in the tourniquet group vs. 4.14 days in the non-tourniquet group, *p*=0.65). There was only 1 thromboembolic event (ischaemic stroke) in the tourniquet group, but the index of suspicion for such events did not differ between cohorts (*p*=0.84). The mean time the tourniquet was inflated was 57.1min (median 60, IQR=45.8−75).

**Table 2:** Primary aim (outcomes).

	Tourniquet	No tourniquet	*P* value	*U* value	*Z* value
Total blood volume loss (mL)	878.19 (826.96; 618.24–1155.71)	1291.91 (1209.22; 1073.45–1497.29)	0.00*	536.00	−4.539
Day 1 pain score (/10)	4.50 (4; 2.75–6)	4.63 (4; 3–6)	0.85	1136.50	−0.179
Knee ROM (degrees)					
Day 2 extension	7.17 (10; 0–10)	6.15 (10; 0–10)	0.42	986.50	−0.809
Day 2 flexion	76.75 (80; 65–90)	78.15 (80; 70–90)	0.83	1059.00	−0.213
Day 2 lag	19.17 (20; 13.75–25)	20.81 (20; 12.50–30)	0.36	754.50	−0.904
Discharge extension	7.30 (10; 3.75–10)	5.90 (5; 0–10)	0.20	946.00	−1.269
Discharge flexion	88.09 (90; 85–90)	85.85 (90; 80–90)	0.24	960.50	−1.178
Discharge lag	15.62 (15; 10–20)	16.05 (15; 10–20)	0.89	1049.00	−0.134
Length of stay (days)	4.57 (4; 3–5)	4.14 (4; 3–5)	0.65	1100.50	−0.454
Index of suspicion for thromboembolic event	8/54 (15%)	7/43 (16%)	0.84		

The data are presented as the mean (median; IQR), where IQR is the interquartile range.

* Marks a significant difference between groups.

### Secondary aim

Women had a lower day 2 maximum flexion (mean 71.6°) when compared to men (mean 84.7°) from the tourniquet cohort, with *p*=0.02. For both the tourniquet and the non-tourniquet groups, women had a longer LOS compared to men: mean 4.5 days for women versus 3.9 days for men in the non-tourniquet group (*p*=0.03) and mean 5.2 days for women versus 3.6 days for men in the tourniquet group (*p*<0.001). In the non-tourniquet cohort, women had a significantly greater day 2 quadriceps lag (mean 25.3) than men (mean 17), with *p*=0.02. Also, in the tourniquet group, men lost more blood than women (1052mL vs. 758mL, *p*<0.001). No difference was found between genders in either subgroup regarding pain, an extension on day 2 and flexion, extension and quadriceps lag at discharge (Table 3). There was no difference between genders in the tourniquet cohort with regards to age (*p*=0.15) and BMI (*p*=0.21). In the non-tourniquet group, men and women were also not different with respect to age (*p*=0.36) and BMI (*p*=0.52).

**Table 3:** Secondary aim (gender subgroup outcomes).

	Males	Females	*P* value	*U* value	*Z* value
Total blood volume loss (mL)					
Tourniquet	1052.81 (979.16; 786.91–1268.29)	758.13 (684.46; 520.69–959.21)	0.00*	191.00	−2.834
No tourniquet	1361.57 (1209.22; 1080.27–1572.18)	1195.17 (1199.69; 971.41–1443.43)	0.43	193.00	−0.788
Knee ROM (degrees)					
Day 2 flexion					
Tourniquet	84.67 (90; 80–90)	71.56 (72.50; 60–85)	0.00*	169.00	−3.093
No tourniquet	79.33 (80; 70–90)	76.47 (80; 67.50–87.50)	0.46	177.00	−0.724
Day 2 lag					
Tourniquet	20.28 (20; 17.50–26.25)	18.46 (17.50; 11.25–25.00)	0.42	217.00	−0.806
No tourniquet	17 (15; 10–30)	25.29 (30; 20–30)	0.02*	96.50	−2.307
Length of stay (days)					
Tourniquet	3.64 (3; 3–4)	5.22 (4; 4–5)	0.00*	179.00	−3.180
No tourniquet	3.88 (3; 3–4)	4.50 (4.50; 3–6)	0.03*	141.50	−2.105

For clarity, only the outcomes that are significantly different between subgroups are presented in the table.

The data are presented as the mean (median; IQR), where IQR is the interquartile range.

* Marks a significant difference between groups.

## Discussion

### Study aims

The literature is quite divisive on how tourniquet use during primary TKA can affect blood loss and functional outcomes. A summary of the most important studies contributing to the debate is presented in Table 4. Our study shows that using a tourniquet can lead to 32% less perioperative blood loss, with similar early postoperative pain, ROM, quadriceps lag, and LOS to tourniquet-less surgery.

**Table 4:** Literature debate summary.

Blood loss	Pro-tourniquet	Cai et al. [18] – Tourniquet use can significantly decrease intraoperative blood loss but did not significantly decrease postoperative blood loss.
	Anti-tourniquet	Schnettler et al. [8] – Tourniquet use with tranexamic acid leads to more blood loss than tranexamic acid alone.
Functional outcomes	Pro-tourniquet	Goel et al. (randomized controlled study) [9] – Tourniquet use has similar in-hospital functional results compared to no tourniquet McCarthy et al. [20] – No clinically important differences in pain, ROM, and LOS during an in-hospital stay between groups.
	Anti-tourniquet	Huang et al. (randomized controlled study) [21] – No tourniquet and tranexamic acid leads to less pain, less swelling, better ROM, and satisfaction during hospital stay compared to tourniquet use Chen et al. (randomized controlled study) [22] – Half-course tourniquet use leads to less pain and better ROM than full-course tourniquet use.
Gender-based outcomes	There is a difference	O’Conner [15] – Gender has been shown to impact both function and pain relief both before and after TKA. Women have a worse preoperative physical function and do not reach the same final level of physical function as men.
	There is no difference	Gen et al. [16] – Gender does not seem to affect short-term outcomes.

Our findings seem to be consistent with a recent meta-analysis showing that tourniquet use can significantly reduce blood loss [18]. Regardless, the results of Schnettler et al. report a paradoxical higher blood loss when using a tourniquet and tranexamic acid compared to using tranexamic acid alone [8]. The blood loss reduction in our study was clinically significant as the only two patients who needed a transfusion were in the non-tourniquet group, but overall the literature seems to suggest that using a tourniquet does not reduce the need for transfusions [18, 19].

Functional results similar to ours have been reported by Goel et al. and McCarthy et al. who showed that there is no differences in pain, ROM and quadriceps function at any point during the in-hospital period between tourniquet and tourniquet-less surgery [9, 20]. By contrast, Huang et al. reported that tourniquet use was associated with lower ROM and higher pain up until the 5th postoperative day [21]. Similarly, Chen et al. demonstrated that tourniquet use for the entirety of the TKA leads to higher pain levels up until postoperative day 3 when compared to patients who had the tourniquet used only during osteotomies [22].

We only had one thromboembolic event (ischemic stroke) in the tourniquet group, but drawing a conclusion from this would be spurious. Most studies dealing with complications after tourniquet use investigate the DVT incidence, but less so the incidence of pulmonary embolism or stroke. Still, some studies suggest there might not be a higher risk for a cumulated thromboembolic event when using a tourniquet [23]. In our study, the number of thromboembolic-related paraclinical procedures did not significantly differ between groups suggesting that neither one was superior at reducing costly investigations.

Gender differences in TKA have been of interest to the orthopaedic community, leading to gender-specific implants. If on this topic the literature seems to point that there is no clinical benefit from gender-specific implants [24] and data on gender-specific outcomes after tourniquet use is sparse and divisive. Some studies claim that women have lower postoperative functional scores than men [15], while others argue that there is no difference [16]. Our data suggest that there might be a difference in how genders tolerate a tourniquet. The blood loss difference in the tourniquet group (1052mL for men vs. 758mL for women), although statistically significant, can be clinically insignificant and could be explained by the different blood volumes between genders. Women had worse outcomes when using a tourniquet, having a day 2 postoperative knee flexion on average 14 degrees lower than men from the same group. Comparatively, in the no tourniquet group, men had a lower day 2 quadriceps lag than women (17 vs. 25.29°). At discharge, these gender-specific functional outcomes did not differ. As there were no differences in pain between males and females in the tourniquet group (mean males pain score 4.91, in females 4.22, *p*=0.43) that could explain an impeded maximum flexion, limb ischemia, and muscle edema may affect women more because of reduced muscle mass. Dreyer showed an accelerated reduction of up to 14% of the quadriceps volume for up to 2 weeks after TKA was performed with a tourniquet [12]. With age, lower limb muscle mass, strength, and gait speed seem to have a faster decline in men, but women lose more quadriceps muscle mass (81% decrease in women vs. 65% in men) [25, 26]. More so, decreased quadriceps muscle strength has been associated with increased mortality risk in the elderly [27].

### Limitations

The primary weakness of this study is that the groups are non-controlled. Secondly, previous anticoagulation treatments were not covered when studying blood loss. Thirdly, our groups had marginally differed age, with the tourniquet group having older people (median of 71years vs. 67years). Although these limitations can be mitigated by all TKAs being performed at a single institution, with identical multimodal perioperative care that includes analgesia and rehabilitation, ultimately each cohort had a different surgeon and implant. Both surgeons were fellowship-trained, high-volume arthroplasty surgeons, though, and used the same standardized methodology when performing the operations, including the same route and dose of tranexamic acid.

## Conclusion

This retrospective cohort study suggests that using a tourniquet during TKA can limit blood loss with no differences in early postoperative functional outcomes, pain, and thromboembolic events compared to tourniquet-less surgery. There may be differences in how genders tolerate tourniquets, with women showing worse short-term functional outcomes. To this extent, we believe there is room for a more tailored approach with a tourniquet being judiciously used according to a patient’s gender, age, biology, and potential muscle mass.

## Conflict of interest

The authors declare that they have no conflict of interest.

## References

[1] Berry DJ, Bozic KJ (2010) Current practice patterns in primary hip and knee arthroplasty among members of the american association of hip and knee surgeons. J Arthroplasty 25, 2–4. 20580196 10.1016/j.arth.2010.04.033

[2] Davies F, Pickford M, Carter D, et al. (2004) National Joint Registry for England and Wales … making it work. NJR 2004. www.njrcentre.co.uk.

[3] Zhang W, Li N, Chen S, et al. (2014) The effects of a tourniquet used in total knee arthroplasty: A meta-analysis. J Orthop Surg Res 9, 13. 24602486 10.1186/1749-799X-9-13 PMC3973857

[4] Yi S, Tan J, Chen C, et al. (2014) The use of pneumatic tourniquet in total knee arthroplasty: A meta-analysis. Arch Orthop Trauma Surg 134, 1469–1476. 25128975 10.1007/s00402-014-2056-y

[5] Alcelik I, Pollock RD, Sukeik M, et al. (2012) A comparison of outcomes with and without a tourniquet in total knee arthroplasty: A systematic review and meta-analysis of randomized controlled trials. J Arthroplasty 27, 331–340. 21944371 10.1016/j.arth.2011.04.046

[6] Rathod P, Deshmukh A, Robinson J, et al. (2015) Does tourniquet time in primary total knee arthroplasty influence clinical recovery? J Knee Surg 28, 335–342. 25180797 10.1055/s-0034-1388654

[7] Ejaz A, Laursen AC, Kappel A, et al. (2014) Faster recovery without the use of a tourniquet in total knee arthroplasty. Acta Orthop 85, 422–426. 24954487 10.3109/17453674.2014.931197 PMC4105775

[8] Schnettler T, Papillon N, Rees H (2017) Use of a tourniquet in total knee arthroplasty causes a paradoxical increase in total blood loss. J Bone Joint Surg Am 99, 1331–1336. 28816892 10.2106/JBJS.16.00750

[9] Goel R, Rondon AJ, Sydnor K, et al. (2019) Tourniquet use does not affect functional outcomes or pain after total knee arthroplasty: A prospective, double-blinded, randomized controlled trial. J Bone Joint Surg Am 101, 1821–1828. 31626006 10.2106/JBJS.19.00146

[10] Pfitzner T, von Roth P, Voerkelius N, et al. (2016) Influence of the tourniquet on tibial cement mantle thickness in primary total knee arthroplasty. Knee Surg Sports Traumatol Arthrosc 24, 96–101. 25248311 10.1007/s00167-014-3341-6

[11] Tai T-W, Chang C-W, Lai K-A, et al. (2012) Effects of tourniquet use on blood loss and soft-tissue damage in total knee arthroplasty: A randomized controlled trial. J Bone Joint Surg Am 94, 2209–2215. 23318610 10.2106/JBJS.K.00813

[12] Dreyer HC (2016) Tourniquet use during knee replacement surgery may contribute to muscle atrophy in older adults. Exerc Sport Sci Rev 44, 61–70. 26829246 10.1249/JES.0000000000000076 PMC4795986

[13] Ricciardi BF, Oi KK, Daines SB, et al. (2017) Patient and perioperative variables affecting 30-day readmission for surgical complications after hip and knee arthroplasties: A matched cohort study. J Arthroplasty 32, 1074–1079. 27876255 10.1016/j.arth.2016.10.019

[14] Mori N, Kimura S, Onodera T, et al. (2016) Use of a pneumatic tourniquet in total knee arthroplasty increases the risk of distal deep vein thrombosis: A prospective, randomized study. Knee 23, 887–889. 27372555 10.1016/j.knee.2016.02.007

[15] O’Connor MI (2011) Implant survival, knee function, and pain relief after TKA: Are there differences between men and women? Clin Orthop Relat Res 469, 1846–1851. 21267799 10.1007/s11999-011-1782-5 PMC3111790

[16] Gen LY, Bin Abd Razak HR, Chi CH, Chye TH (2015) No gender-based differences in outcomes after conventional total knee arthroplasty in Asians. J Arthroplasty 30, 1548–1550. 25869589 10.1016/j.arth.2015.03.021

[17] Magill P, Cunningham EL, Hill JC, Beverland DE (2018) Identifying the period of greatest blood loss after lower limb arthroplasty. Arthroplast Today 4, 499–504. 30569010 10.1016/j.artd.2018.09.002 PMC6288045

[18] Cai DF, Fan QH, Zhong HH, et al. (2019) The effects of tourniquet use on blood loss in primary total knee arthroplasty for patients with osteoarthritis: A meta-analysis. J Orthop Surg Res 14, 1–9. 31703706 10.1186/s13018-019-1422-4 PMC6839231

[19] Smith TO, Hing CB (2010) Is a tourniquet beneficial in total knee replacement surgery? A meta-analysis and systematic review. Knee 17, 141–147. 19616954 10.1016/j.knee.2009.06.007

[20] McCarthy Deering E, Hu SY, Abdulkarim A (2019) Does tourniquet use in TKA increase postoperative pain? A systematic review and meta-analysis. Clin Orthop Relat Res 477, 547–558. 30461513 10.1097/CORR.0000000000000572 PMC6382207

[21] Huang Z, Xie X, Li L, et al. (2017) Intravenous and topical tranexamic acid alone are superior to tourniquet use for primary total knee arthroplasty: A prospective, randomized controlled trial. J Bone Joint Surg Am 99, 2053–2061. 29257010 10.2106/JBJS.16.01525

[22] Chen S, Li J, Peng H, et al. (2014) The influence of a half-course tourniquet strategy on peri-operative blood loss and early functional recovery in primary total knee arthroplasty. Int Orthop 38, 355–359. 24258152 10.1007/s00264-013-2177-x PMC3923944

[23] Li X, Yin L, Chen Z-Y, et al. (2014) The effect of tourniquet use in total knee arthroplasty: Grading the evidence through an updated meta-analysis of randomized, controlled trials. Eur J Orthop Surg Traumatol 24, 973–986. 23842662 10.1007/s00590-013-1278-y

[24] Sappey-Marinier E, Swan J, Batailler C, et al. (2020) No clinical benefit from gender-specific total knee replacement implants: A systematic review. Sicot-J 6, 25. 32618563 10.1051/sicotj/2020023 PMC7333614

[25] Shaw SC, Dennison EM, Cooper C (2017) Epidemiology of sarcopenia: Determinants throughout the lifecourse. Calcif Tissue Int 101, 229–247. 28421264 10.1007/s00223-017-0277-0 PMC5544114

[26] Kasai T, Ishiguro N, Matsui Y, et al. (2015) Sex- and age-related differences in mid-thigh composition and muscle quality determined by computed tomography in middle-aged and elderly Japanese. Geriatr Gerontol Int 15, 700–706. 25244543 10.1111/ggi.12338

[27] Newman AB, Kupelian V, Visser M, et al. (2006) Strength, but not muscle mass, is associated with mortality in the health, aging and body composition study cohort. J Gerontol A Biol Sci Med Sci 61, 72–77. 16456196 10.1093/gerona/61.1.72

